# Biases in human perception of facial age are present and more exaggerated in current AI technology

**DOI:** 10.1038/s41598-022-27009-w

**Published:** 2022-12-29

**Authors:** Tzvi Ganel, Carmel Sofer, Melvyn A. Goodale

**Affiliations:** 1 Department of Psychology, Ben-Gurion University of the Negev, 8410500 Beer-Sheva, Israel; 2 Department of Cognitive and Brain Sciences, Ben-Gurion University of the Negev, 8410500 Beer-Sheva, Israel; 3 Department of Industrial Engineering and Management, Ben-Gurion University of the Negev, 8410500 Beer-Sheva, Israel; 4 The Western Institute for Neuroscience, The University of Western Ontario, London, ON N6A 5B7 Canada

**Keywords:** Human behaviour, Perception, Machine learning

## Abstract

Our estimates of a person’s age from their facial appearance suffer from several well-known biases and inaccuracies. Typically, for example, we tend to overestimate the age of smiling faces compared to those with a neutral expression, and the accuracy of our estimates decreases for older faces. The growing interest in age estimation using artificial intelligence (AI) technology raises the question of how AI compares to human performance and whether it suffers from the same biases. Here, we compared human performance with the performance of a large sample of the most prominent AI technology available today. The results showed that AI is even less accurate and more biased than human observers when judging a person’s age—even though the overall pattern of errors and biases is similar. Thus, AI overestimated the age of smiling faces even more than human observers did. In addition, AI showed a sharper decrease in accuracy for faces of older adults compared to faces of younger age groups, for smiling compared to neutral faces, and for female compared to male faces. These results suggest that our estimates of age from faces are largely driven by particular visual cues, rather than high-level preconceptions. Moreover, the pattern of errors and biases we observed could provide some insights for the design of more effective AI technology for age estimation from faces.

There is little doubt that human observers can readily extract visual information from faces to estimate a person’s age^1^. Cues from wrinkling, pigmentation, hair color and facial structure all contribute to these estimates^2^. Yet, human accuracy in age evaluations is imprecise, limited by the observer’s perceptual resolution, top-down influences^3^, and by genetic and environmental factors that cause people’s faces to age differently^4^.

In the current study, we compared the performance of human observers and several popular Artificial Intelligence (AI) programs in estimating people’s ages from photos of their faces. As it turns out, AI platforms make many of the same errors as humans, although to a larger extent. Before exploring what this might mean, we briefly review the kinds of errors that humans typically exhibit when estimating someone’s age and the potential sources of those errors.

Previous research has identified several well-known biases and inaccuracies in our ability to estimate age from facial appearance^2^. For example, there is a significant decrease in accuracy when estimating age from the faces of middle-aged adults (40–60) compared to young adults (20–40) and of older adults (ages 60–80) compared to middle-aged adults^2,5^. This decrease in accuracy could be accounted for by the fact that genetic and environmental factors have a larger impact on the appearance of the face as people grow older, and that there is considerable variance in these effects on the apparent age of the face^2^. The inaccuracies could also stem, at least in part, from biases in evaluating the age of people of different ages: the faces of young adults (ages 20–40) are typically perceived as older than their actual age, whereas the faces of older adults are typically perceived as younger^2,5^.

These biases are likely due to people’s tendency to use the estimated mean of a given dimensional distribution as reference point for their judgments^6^, a regression to the mean effect. In the case of age evaluation, assuming that the perceived mean age of the population is around 40–45 years, it has been suggested that age evaluations of younger and older adults tend to drift to the direction of this perceived mean, creating a bias in which the age of younger adults is overestimated while age evaluations of older adults are biased in the opposite direction^2^. We note, however, that while there is partial correlation between bias and accuracy measures in age perception, as we discuss below, the regression to the mean effect could not fully account for the general decrease in accuracy performance with the age of the presented faces. In particular, the bias in age estimations for faces of younger (and older) adults is significantly larger than the bias for faces of middle-aged adults^2,5^, but at the same time, the accuracy of age estimations for faces of middle-aged adults is significantly improved compared to the accuracy for faces of young adults^2,5^.

Perhaps one of the most intriguing biases in age evaluation can be seen in the way smiling influences perceived age. Recent research from our lab and others strongly suggests that, contrary to common belief^7^, smiling faces are perceived as older than the faces of the same people when they have a neutral expression^5,7,10^. This “ageing effect of smiling” (AES) is assumed to be driven by the formation of smile-related wrinkles in the region of the eyes. Remarkably, we observed a sharp dissociation between what the participants believed they had reported and how they actually performed. Thus, in the same experimental session, participants estimated smiling faces as looking older than neutral faces, but at the same time, erroneously assumed that they had rated the smiling faces as younger^7,9^.

More recent data shows that the AES is reduced with age of the faces presented. The AES is most prominent for faces of young adults of either gender, is evident only in male faces of middle-aged adults, and completely vanishes for the faces of older adults^5^. This modulation of the AES by the age of the presented faces does not depend on the age of the observer, and is attributed to the relative contribution of information from smile-related wrinkles compared to that provided by other cues to aging. Neutral faces of young adults, which contain few age-related signs, are easily offset by the addition of smile-related wrinkling, while the same is not true for faces of older adults where there are a number of other prominent cues to aging^5^.

The AES represents a basic bias in age evaluations for smiling faces compared to neutral ones. But beyond this bias, age evaluations of smiling faces (as well as faces with any expression) are generally less accurate than age evaluations of neutral faces. In developing this idea, it is important to distinguish between two classic aspects of age evaluation. The first is a bias in perceived age; the second is the absolute accuracy of age evaluation. The bias, by definition, is directional (signed), and is measured by subtracting the actual age from the estimated age of a face. In contrast, the accuracy of age evaluations is measured by computing the absolute differences between the actual and the perceived age. In psychophysical terms, the accuracy in age evaluations is related (but not identical) to the concept of the JND (just noticeable difference) measured by the variability of the response^11,12^, which, in this case, represents the resolution of perceived estimates of age. Bias, however, is related to the psychophysical concept of Constant Error (CE)^13,14^, and represents the directional difference between the perceived and actual age of a given face or subset of faces. It should be noted that while the two aspects of age evaluations are sometimes related, they can also be partly independent from one another. For example, a bias in age evaluations would definitely result in inaccurate performance, but at the same time, a zero bias in age evaluation does not mean that age estimates are accurate. Consider the case in which there is symmetrical, yet substantial variability in age evaluations of a particular face (e.g., the person’s actual age is 40 years old, but she/he is perceived to be 20 by some people and 60 by others). In this case, the bias in age estimation is zero, yet accuracy is extremely poor, with an absolute error of 20 years!

It is important to note that the decrease in accuracy for smiling faces is not solely the result of the AES, but rather represents a more general effect of facial expression on accuracy^2,5^. This is illustrated by the fact that the decrease in accuracy for smiling compared to neutral faces is found even for faces of older adults, for which there is no AES^2,5^. The decrease in performance for smiling faces likely represents difficulty in estimating the age of expressive faces due to temporary variations in the appearance of the face that are part of emotional expression^2^. Previous research has also shown that accuracy in age estimations is higher for male than for female faces^2,5,15^. Yet, unlike the case of expression, the gender-based accuracy effect can be accounted for, at least in part, by biases in age estimation. In particular, the age of female faces is underestimated to a larger degree than the age of male faces, an effect that is more prominent with faces of older adults^2,5^. Given that accuracy differences in gender are also more prominent with faces of older adults^5^, it could be argued that the poorer age-estimation performance for female faces results from this bias.

Recently, there has been a growing interest in automated age estimation using artificial intelligence (AI) technology^16^. The current platforms use machine-learning algorithms based on training with a large set of photos to achieve the most accurate performance in age estimations. The current interest in age estimation by AI is part of an overall attempt to extract various visual features automatically from faces, features that include identity, expression, and gender as well as other information that can be gleaned from the face. The specific interest in age estimation is also boosted by recent commercial incorporation of automatic age estimation technology for different uses, including age verification in retail outlets that is now being implemented in different countries^17^. Beyond dozens of commercial companies that offer age estimation technologies, there are also many non-commercial apps and webpages that offer age estimation technology based on photos uploaded by the users. Despite this growth in the technology, it is presently unclear how AI compares to human performance and whether it suffers from the same biases and errors. Here, we provide the most comprehensive attempt to date comparing age estimation performance between humans and the most popular AI technology available today.

Comparing human performance with the performance of AI could provide a better understanding of the processes that underlie age perception in humans. In particular, it allows one to tease apart possible top-down cognitively driven effects from those driven by the image itself. For example, although the ageing effect of smiling (AES) has been attributed to wrinkling around the eyes, it is also possible that this effect is modulated by people’s general belief that smiling makes one look younger, and without this modulation, the AES would be even larger^7,8^. In a related manner, although the decrease in the accuracy of age estimation for older adults has been traditionally attributed to the visual properties of faces, it could also be due to the common belief that accuracy in age estimations for faces of older adults is a less critical issue than accuracy for young adults, and therefore should be taken more lightly^2^. In sum, to the extent that AI suffers from the well-established biases and inaccuracies that are part of human performance, it could be argued that the biases commonly exhibited by human observers are largely driven by the visual properties of faces.

Beyond the focus on the factors driving human perception of age, the comparison between human and AI abilities could provide a better understanding of current AI technology and suggest ways to improve this technology. There is strong evidence, for example, that, for other aspects of face recognition such as face identity and gender classifications, current AI technology shows discrimination biases based on ethnicity, gender, and age^18,19^. It is unclear whether and to what extent, compared to humans, automatic age estimation technology suffers from similar biases. Here, we used a comprehensive sample of 21 different AIs, including commercial programs such as Microsoft Face API, Amazon Rekognition, Everypixel API, as well as leading non-commercial websites and applications (for a full list, see Table 1). We compared AI performance to the performance of human observers using a large set of neutral and smiling female and male faces from different age groups that we have previously reported^5^.

**Table 1:** Description of the different AIs tested for age evaluations.

	Name of AI algorithm	Website	Data acquisition date	Comments
AI1	Microsoft Face API	https://azure.microsoft.com/en-in/services/cognitive-services/face/	11/20	
AI2	“How old do I look” website (by Microsoft)		4/20	Discontinued (2020)
AI3	Everypixel API	https://labs.everypixel.com/api/demo	11/21	
AI4	Visage Technologies Face detector	https://www.visagetechnologies.com/HTML5/latest/Samples/ShowcaseDemo/ShowcaseDemo.html#	12/21	
AI5	Sightcorp F.A.C.E. API	https://face-api.sightcorp.com/demo-basic/	12/21	
AI6	howolddoyoulook.com	https://howolddoyoulook.com/	1/22	
AI7	FacialAge Detect age by photo	https://www.facialage.com/	2/22	
AI8	eydea EyeFace	https://cloud.eyedea.cz/api/face	2/22	
AI9	VeriLook Face SDK	https://www.neurotechnology.com/verilook.html	2/22	
AI10	AgeGuesser	https://imageamigo.com/age/	4/22	
AI11	Amazon Rekognition	https://docs.aws.amazon.com/rekognition/latest/dg/what-is.html	2/22	
AI12	Estimate My Age	https://apps.apple.com/us/app/estimate-my-age-face-app/id1028912349	3/22	Appstore app
AI13	How Old Do I Look (app)	https://apps.apple.com/us/app/how-old-do-i-look/id1442557104	3/22	Appstore app
AI14	Betaface API	https://www.betafaceapi.com/demo.html	3/22	
AI15	FACE++	https://www.faceplusplus.com/attributes/	3/22	
AI16	Facelytics face recognition	https://www.facelytics.io/	3/22	
AI17	FaceX	https://facex.io/	5/22	
AI18	AgeBot	https://play.google.com/store/apps/details?id=com.testa.agebot&hl=iw&gl=US	3/22	Play store app
AI19	Check My Age	https://play.google.com/store/apps/details?id=com.neurotec.checkmyage&hl=iw&gl=US	3/22	Play store app
AI20	Reconess	https://reconess.com/products/analysis	3/22	
AI21	Shore	https://www.iis.fraunhofer.de/en/ff/sse/affective-computing/facial-analysis-solutions/download.html	3/22	

## Methods

### Participants

The data of AI performance was collected over the years 2020–2022, providing a representative set of 21 current commercial and non-commercial AI age estimation technology (see Table 1). We did our best to include the most prominent players in the field as well as the most popular available websites and apps. Although it is possible that we missed some, we believe that our survey includes a comprehensive sample of current AI technology. AI performance was compared with the performance of 30 undergraduate students from Ben Gurion University of the Negev (12 males, mean age = 23.4 years, SD = 1.63 years), originally reported in Ganel and Goodale (2021), and reanalyzed for the purpose of the current study. The experimental protocol was approved by the ethics committee of the Department of Psychology in Ben-Gurion University of the Negev. The study adhered to the ethical standards of the Declaration of Helsinki. All participants signed an informed consent form prior to their participation in the experiment. The manuscript contains no information or images that could lead to identification of a study participant.

### Stimulus set and design

**Figure 1:**
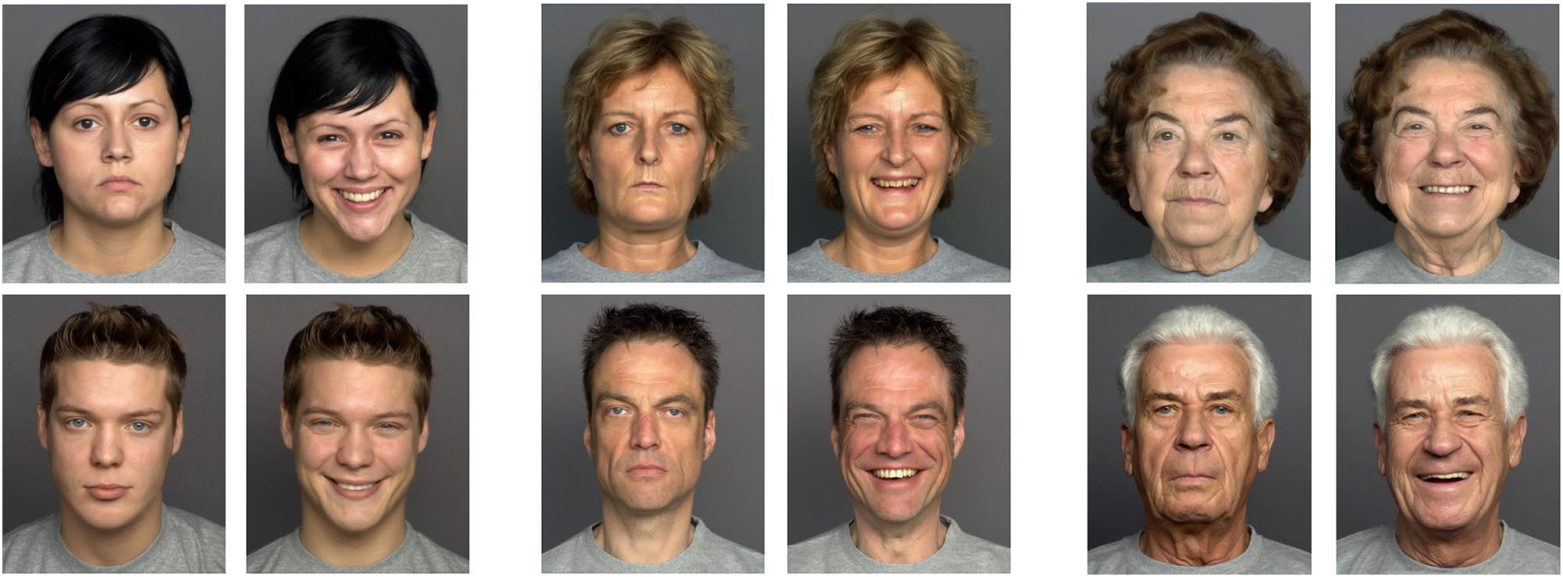
Examples of stimuli used in the study. The images were of neutral and smiling faces of young adults, middle-aged adults, and old adults. Adapted from Ebner et al.^20^, all right reserved.

The stimulus set was identical to the one recently used in our lab^5^. The set comprised 480 photos of women and men, each photographed with neutral and smiling expressions. The set was based on three databases that included the real ages of the photographed people: The FACES database^20^, the PAL face database^21^, and a set of faces photographed by members of the Ganel lab. The photos were equally divided into 3 age groups: young adults (20–40 years), middle-aged adults (40–60), and old adults (60–80 years). Photos were in .bmp format (240–357 × 300 pixels, depending on the source set, 24-bit depth). Figure 1 shows examples of photos used in the set. For full description of the set, see Ref.^5^.

Each photo was uploaded separately for age evaluations by the different AIs and the output was recorded in years. For two of the AIs (AI11, AI16) that provided output age range instead of a 2-digit output, the average of the range was used. Photos were uploaded in a .bmp format. In cases in which .bmp was not supported by the AI, high quality .jpeg versions of the photos with similar dimensions were uploaded. Note that 5 of the AIs (AI3, AI9, AI14, AI18, AI19) did not provide age outputs larger than 69 years of age. While this limitation could be seen as the first indication of “ageism” in AI, a trend which also appeared in the main analysis and will be discussed in the following sections, the general pattern of results and biases was maintained even when the data of these specific AIs were excluded in a separate analysis.

The full experimental procedure for the human participants is described in Ref.^5^. As in the case of the AIs, each photo was presented separately, and participants were asked to type their two-digit response on the keyboard. The only substantial difference in the design was that in order to prevent top-down memory influences for human participants, the stimulus set was counterbalanced so that each participant was presented with only one photo (smiling or neutral) of the same person^5,7–10^. This concern was irrelevant in the case of AIs, because none of them stored previously presented data or relied on previous responses for age estimations, which were based entirely on the presented image. Therefore, the entire set of 480 faces was presented to each of the AIs. To assure reliability, we also randomly uploaded some of the photos twice and on different occasions for a sample of the AIs. In all these cases, the outputs were identical.

### Analysis

For each human participant/AI, accuracy scores were computed for each photo using the average absolute difference between the estimated and real age. Bias scores were computed by subtracting the real age from the estimated age. For each participant and for each AI, we computed the average estimated age, the mean accuracy score, and the mean bias score in each combination of age group X gender X expression. The descriptive data in all tables and figures were based on this analysis. In order to compare human and AI performance statistically, we reanalyzed the data in an item-based manner for which each presented person (photo) was treated as separate item. 240 items were therefore included in this analysis. Expression (neutral, smiling) for each item was treated as a within-item variable. Data was then averaged separately across all human participants and across all AIs for each expression and for each photo. A mixed ANOVA design was used to analyze the data, in which the expression of the photographed person (smiling, neutral with 240 photos in each group) and the experimental group (humans, AIs) were the within-subject (item) variables, and the gender of the photographed person and his or her age group were between-subjects (items) variables. During the item analysis, we found that the identity of one of the 480 photos belonged to a different person than intended. This particular photo was removed from all subsequent analyses.

## Results

Table 2 presents the accuracy scores for each combination of gender, expression, and age group for each of the AIs. Average human performance in each combination is presented for reference. Table 3 presents average estimated ages in each combination for each of the AIs, the average perceived age for humans, and the average real age.

**Table 2:** Absolute age estimation accuracy (in years) of the different AIs compared to average human performance.

	Young adults				Middle-aged adults				Old adults			
	Female faces		Male faces		Female faces		Male faces		Female faces		Male faces	
	Neutral	Smiling	Neutral	Smiling	Neutral	Smiling	Neutral	Smiling	Neutral	Smiling	Neutral	Smiling
AI1	3.90	6.33	4.35	5.35	2.93	3.38	3.25	3.43	9.95	10.56	5.40	5.65
AI2	5.13	8.15	5.63	7.53	7.43	7.40	6.30	8.03	10.72	10.08	6.20	6.70
AI3	3.33	4.10	2.78	3.20	4.80	4.98	4.30	4.78	8.72	8.92	10.28	11.50
AI4	4.03	4.50	3.38	4.73	3.68	4.20	3.15	4.45	4.46	5.41	4.13	5.65
AI5	6.80	9.53	7.05	8.33	5.83	7.98	4.78	5.83	14.44	16.15	10.33	13.23
AI6	3.72	5.91	3.52	5.60	5.30	6.48	3.75	6.01	5.21	5.17	4.56	4.22
AI7	7.18	11.13	6.90	9.43	4.63	4.73	3.48	3.70	10.54	12.51	7.35	8.15
AI8	5.70	5.53	4.30	4.33	4.10	5.28	4.18	5.20	10.49	10.46	5.78	6.15
AI9	3.40	4.15	2.88	2.83	4.40	6.80	4.88	6.65	9.87	12.59	6.60	10.78
AI10	3.18	4.90	2.98	4.23	3.44	3.36	4.83	5.35	5.45	7.55	4.83	7.10
AI11	3.48	6.93	2.98	4.53	3.33	3.15	3.63	3.08	6.46	9.15	6.10	7.75
AI12	4.40	6.78	4.60	5.80	3.38	3.28	4.68	4.80	9.87	10.62	5.40	5.55
AI13	3.70	4.80	4.33	4.88	5.93	8.05	6.03	7.98	7.13	7.59	6.73	6.43
AI14	5.70	9.58	7.20	12.05	10.18	10.58	5.40	7.05	21.38	22.56	15.90	16.53
AI15	6.63	16.25	3.95	9.25	7.83	14.55	6.95	11.85	4.26	7.08	4.30	7.00
AI16	8.08	11.85	4.70	8.95	3.85	2.85	2.38	2.53	8.40	9.65	8.90	10.78
AI17	2.30	3.18	2.60	3.00	5.83	6.68	4.65	4.13	14.95	15.31	10.28	10.53
AI18	4.48	6.63	4.35	4.83	5.00	6.70	3.70	3.95	4.15	6.72	4.58	5.58
AI19	3.38	4.60	2.78	2.98	4.30	6.78	4.80	6.65	10.03	12.03	6.60	10.68
AI20	3.40	5.20	3.03	4.88	5.53	4.35	4.20	4.38	4.77	7.95	5.08	8.08
AI21	5.08	5.48	5.98	7.10	10.98	10.80	7.75	8.13	12.54	18.08	8.78	9.23
Avg AI	4.62	6.93	4.30	5.89	5.36	6.30	4.62	5.61	9.23	10.77	7.05	8.44
Avg. humans	4.46	5.03	4.42	5.32	6.32	6.82	6.44	6.60	7.34	7.89	6.77	6.65

**Table 3:** Average estimated age (in years) for the different AIs compared to average human performance.

	Young adults				Middle-aged adults				Old adults			
	Female faces		Male faces		Female faces		Male faces		Female faces		Male faces	
	Neutral	Smiling	Neutral	Smiling	Neutral	Smiling	Neutral	Smiling	Neutral	Smiling	Neutral	Smiling
AI1	28.43	31.15	28.80	29.60	48.65	48.30	49.08	49.85	61.51	60.85	66.53	66.03
AI2	26.85	31.08	29.98	31.73	46.05	48.38	48.08	52.47	64.54	65.03	70.68	71.78
AI3	25.50	27.68	24.53	25.25	46.23	45.30	45.13	45.15	62.85	62.74	61.20	59.98
AI4	22.55	24.68	25.23	26.88	51.10	52.48	48.33	51.28	68.69	68.00	68.65	66.48
AI5	30.98	33.05	31.15	31.63	48.50	47.50	47.15	46.00	56.92	55.62	61.55	58.70
AI6	23.73	29.59	25.99	28.37	52.32	55.49	49.11	53.17	70.68	70.05	70.35	70.42
AI7	32.10	36.05	31.75	34.33	45.50	45.60	45.75	47.73	60.77	58.79	64.38	63.63
AI8	28.03	26.20	26.80	26.43	48.58	50.35	47.85	47.78	70.70	68.84	67.10	66.43
AI9	23.68	25.23	24.28	24.03	49.58	44.33	46.75	44.43	61.54	58.72	64.88	60.70
AI10	23.10	22.73	24.88	23.08	48.31	48.69	45.25	45.18	67.76	67.03	66.85	65.03
AI11	27.85	31.20	26.08	28.43	49.55	49.98	47.80	49.00	66.49	62.67	66.13	64.18
AI12	28.78	31.60	29.15	30.15	48.55	48.80	49.28	50.03	61.49	60.90	66.48	66.23
AI13	27.93	28.18	27.93	27.88	54.00	57.08	53.20	55.10	77.56	77.41	78.10	77.05
AI14	24.88	33.20	28.05	34.45	40.80	42.40	45.23	47.68	49.92	48.74	55.58	54.95
AI15	30.30	41.08	26.15	33.20	57.35	64.38	54.68	60.18	73.77	77.92	74.98	78.48
AI16	32.53	35.98	28.40	32.90	46.78	49.35	46.43	48.20	62.91	61.68	62.60	60.73
AI17	25.18	27.25	25.10	25.80	44.10	43.45	43.83	44.90	56.36	56.00	61.20	60.95
AI18	27.30	28.25	26.45	27.78	52.13	54.13	48.68	50.13	70.54	69.97	68.75	67.95
AI19	23.55	25.28	24.28	24.58	49.53	44.30	47.13	44.63	61.28	59.28	64.88	60.80
AI20	25.98	29.63	25.38	28.43	52.50	50.48	48.83	48.00	72.69	68.49	68.20	64.85
AI21	23.20	28.75	30.03	28.90	45.30	41.18	50.98	50.30	59.33	53.38	64.60	63.20
Avg AI	26.78	29.89	27.16	28.75	48.83	49.14	48.02	49.10	64.68	63.43	66.36	65.17
Avg. humans	26.92	28.11	26.25	27.19	48.55	48.58	48.75	49.87	67.75	67.32	68.56	68.98
Avg. real age	24.93		24.95		49.83		48.38		71.30		71.48	

**Figure 2:**
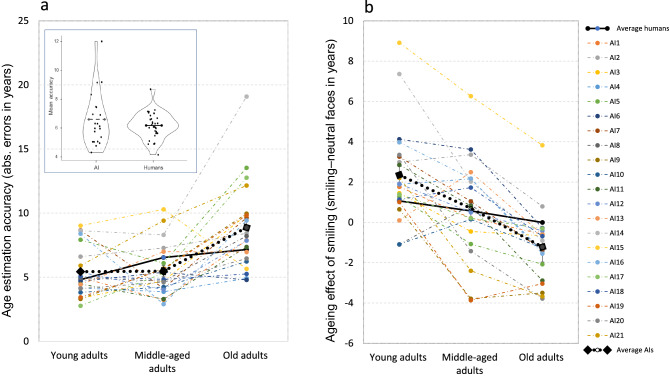
(**a**) Age estimation accuracy for the different AIs compared to average human performance. As was the case for human observers, AI performance showed a large decrease in accuracy for faces of older adults compared to faces of younger adults. This effect was larger for AI compared to human performance. (**b**) The ageing effect of smiling for the different age groups—smiling faces were estimated as older than neutral faces of the same people. Overall, this effect was larger for AI (younger adults), and showed a sharper decrease with age. The inset shows the mean accuracy data (across age group, gender and facial expression) for each AI and each of the human observers. Group averages are indicated by the horizontal lines. Although there was variability in age estimation accuracy in both groups, humans were less variable and more accurate.

As can be seen in the tables, errors and biases across the AIs were similar to the well-established errors and biases found for human perception. First, average AI performance sharply decreased for faces of older adults compared to faces of young and middle-aged adults. As illustrated in Fig. 2a (average accuracy scores in each age group), this decrease with age was larger than the decrease observed in human performance. Second, and perhaps more interesting, is the fact that just as in human perception, AI estimated the ages of smiling faces as older than the neutral faces of the same people (Fig. 2b). As was the case for human observers, this effect was most pronounced for faces of younger and middle-aged adults, and decreased for faces of older adults. However, the size of the AES for AI was larger for faces of young adults compared to the AES seen in human observers with the faces of young adults, and the decrease in the size of the AES for older faces was also larger and went in the opposite direction for AI. It is important to note, however, that there was considerable variability in the performance of the 21 AI programs we tested. Nevertheless, as the inset in Fig. 2 shows, variability was also evident in our human participants, although to a lesser degree.

**Figure 3:**
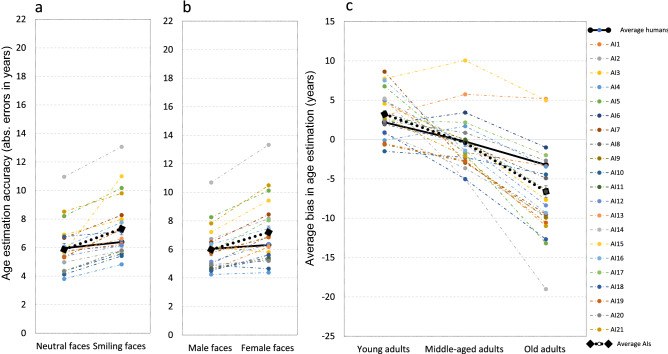
(**a**) Average age estimation accuracy for neutral and smiling faces across age group and gender. As in human performance, AI accuracy decreased for smiling compared to neutral faces. This decrease was larger for AI compared to humans (**b**) Average age estimation accuracy for male and female faces across age group and expression. As for humans, AI age estimation accuracy decreased for female compared to male faces. This decrease was larger in AI. (**c**) Age estimation bias for the different AIs across expression and gender. As in human age estimations, AI showed overestimation of age for faces of younger adults and underestimation of age for faces of older adults. Again, this effect was more pronounced in AI compared to humans.

A similar trend of human-like biases in AI age estimation (although to a higher degree) was found for the effect of expression and gender on accuracy (Fig. 3a,b). As was the case for human observers, AI performance decreased for smiling compared to neutral faces, an effect that was evident across all age groups. Again, this decrease in accuracy was larger for AI than it was for human observers. A similar trend was found for the effect of gender. Performance accuracy for male faces was higher than for female faces, an effect found for all age groups in AI but only for old adults in the human observers (Table 3). Not surprisingly, therefore, the overall decrease in age accuracy for female compared to male faces was larger in AI (Fig. 3b). Finally, as in the case of human performance, AI showed age-group dependent biases. In particular, faces of young adults were overestimated compared to their real age while faces of older adults were underestimated compared to their real age. Again, this modulation of the bias with age group was larger for AIs compared to human age estimation (Fig. 3c).

### Statistical comparison between AI and human age estimations

To compare AI and human age estimations, we used an item-based analysis in a mixed ANOVA design with expression (smiling, neutral), and experimental group (humans, AIs) as the within-subject (item) variables and with gender and age group as between-subjects (items) variables. The dependent variables were the absolute accuracy score, the mean estimated age, and the directional bias in age estimations.

For accuracy, there was a main effect of group [F(1,233) = 8.27, p < 0.001, η_p_^2^ = 0.07], indicating that the performance of the human observers was more accurate overall than the performance of AI (see Fig. 2). We will discuss this effect in the next section. A main effect of age group [F(1,233) = 18.27, p < 0.001, η_p_^2^ = 0.07] indicated an overall drop in performance with the faces of older compared to young and middle-aged adults [Fig. 2a; F(2,233) = 68.02, p < 0.001, η_p_^2^ = 0.37]. This main effect was qualified by a significant interaction between group (AI and human observers) and age group [F(1,233) = 50.78, p < 0.001, η_p_^2^ = 0.3], which indicates that the decrease in accuracy with age group was larger for AIs than it was for human observers. A main effect was also found for the gender of the photo [F(1,233) = 13.28, p < 0.001, η_p_^2^ = 0.05], indicating a decrease in accuracy for female faces. Again, the main effect was qualified by significant gender X group (AI and human observers) interaction [F(1,233) = 16.57, p < 0.001, η_p_^2^ = 0.06], showing that the decrease in performance for female faces was larger in AI (see Fig. 3b). A main effect was also found for expression [F(1,233) = 122.32, p < 0.001, η_p_^2^ = 0.34], indicating reduced accuracy for smiling compared to neutral faces. Again, this main effect was qualified by expression X group (AI and human observer) interaction [F(1,233) = 60.59, p < 0.001, η_p_^2^ = 0.16], again showing that the effect of expression on accuracy was larger in AI (Fig. 3a).

An interaction between expression and age group [F(2,233) = 5.59, p < 0.01, η_p_^2^ = 0.05] indicated that the effect of expression was more pronounced in the faces of young adults compared to the other age groups. A gender X age-group interaction [F(2,233) = 4, p < 0.05, η_p_^2^ = 0.03] indicated that the effect of gender was larger in the old adults group compared to the other groups. All other interactions were not significant (p > 0.05).

For the dependent variable of average estimated age, there was a main effect of expression [F(1,233) = 13.96, p < 0.001, η_p_^2^ = 0.06], indicating that overall, smiling faces were estimated as older than neutral faces (the AES). This main effect was qualified by an expression X age group interaction [F(2,233) = 20.13, p < 0.001, η_p_^2^ = 0.15], indicating a decrease in the AES with age group. This decrease was larger for AIs compared to human observers, as indicated by a significant group (AI and human observers) X expression X age-group interaction [F(1,233) = 12.74, p < 0.001, η_p_^2^ = 0.1].

A main effect of group (AI and human observer) [F(1,233) = 16.78, p < 0.001, η_p_^2^ = 0.07] indicated that, overall, age was underestimated by the AIs compared to human observers. A main effect of age group [F(2,233) = 1552.46, p < 0.001, η_p_^2^ = 0.93] indicated older age estimations for older adults. This effect was qualified by group X age group interaction [F(2,233) = 47, p < 0.001, η_p_^2^ = 0.29], indicating that faces of older adults were more strongly underestimated by AIs compared to humans than in the other age groups. A marginally significant 3-way interaction between group (AI and human observer), expression, and gender [F(1,233) = 4.28, p = 0.04, η_p_^2^ = 0.02], probably resulted from the larger decrease in performance for male smiling faces compared to female smiling faces in human, but not AI, estimations of age. All other main effects and interactions were not significant (p > 0.05).

Finally, we analyzed the dependent variable of bias in age estimation using a similar design. We note that there is an overlap between this analysis and the analysis of average perceived age. However, because the differences between the estimated and the real ages within each combination of age group and gender were not identical, we decided to include this analysis as well. There was a main effect of group [F(1,233) = 18.94, p < 0.001, η_p_^2^ = 0.08], indicating that, overall, AI underestimated the age of faces more than human observers did. A main effect of age group [F(2,233) = 101.2, p < 0.001, η_p_^2^ = 0.47] indicated that the bias in age estimations was modulated by age. A group (AI vs. human observer) X age-group interaction [F(2,233) = 47.73, p < 0.001, η_p_^2^ = 0.29], showed that age overestimation of faces of younger adults and underestimation of faces of older adults were larger in AI (Fig. 3c).

A main effect of expression [F(1,233) = 13.96, p < 0.001, η_p_^2^ = 0.06] indicated that smiling faces were estimated to be older than neutral faces (the AES). This effect was qualified by an expression X age-group interaction [F(2,233) = 20.1, p < 0.001, η_p_^2^ = 0.15], suggesting that there was a decrease in AES as people got older. A 3-way group X expression X age-group interaction [F(2,233) = 12.7, p < 0.001, η_p_^2^ = 0.09] indicated that the decrease in AES with age group was larger in AI. A 3-way group (AI vs. human observer) X expression X gender interaction [F(2,233) = 4.3, p < 0.05, η_p_^2^ = 0.02] probably resulted from the fact that the interaction between gender and expression was larger for human observers than it was for AIs. All other main effects and interactions were not significant (p > 0.05).

## Discussion

The general pattern of results was robust: AI showed human-like age estimation biases and inaccuracies across all aspects of performance tested in the current study. Moreover, all biases and inaccuracies were significantly larger in AI than in human observers.

Because AI programs, unlike human observers, have no top-down preconceptions or opinions about the effects of age range, gender, or facial expression on apparent age, the results suggest that the well-established biases in human age perception are heavily driven by the visual properties of the faces. In the case of the ageing effect of smiling (AES), the results reinforce the notion that smiling faces are estimated as looking older than neutral faces of the same individuals because of the formation of smile-related wrinkles around the eyes^8^. As in human perception, the AES in AI was demonstrated for faces of young and middle-aged adults. Still, for faces of young adults, the AES was on average more than two-fold larger in AI compared to human observers (2.4 vs. 1.1 years) and was also moderately larger for faces of middle-aged adults (0.7 vs. 0.6 years).

Interestingly, for faces of older adults, for which AES is absent in human perception^5^, AI showed an opposite pattern of results: smiling faces were perceived as younger than neutral faces. We can only speculate as to the source of this effect. Recently, we observed a similar pattern of results in human observers who were asked to estimate the age of photos of faces of old adults wearing face masks^22^. The similarity between patterns of results may imply that machine-learning technology relies more heavily on partial facial information in the upper region of the face that is unobscured by masks^23^. The use of such information has also been associated with reduced holistic processing of faces^23–25^.

The accuracy of age estimation by AIs showed a significant decrease for faces of older adults compared to faces of young and middle-aged adults. Such a decrease in performance has been previously observed in human age perception, and results, at least in part, from an objective difficulty in estimating the age of older adults^2^. Yet, this decrease is larger in AIs. The large difference in accuracy between human observers and AI for faces of older adults compared to faces of other age groups cannot be easily accounted for by the objective difficulty of extracting age from face photos. After all, a few of the AIs we tested performed particularly well, even for faces of older adults (Fig. 2a), which suggests that potentially, visual information can be used effectively to achieve reasonable performance. In short, there is an indication of “ageism” in some AIs, which could be due to inequalities along the age range of the sets of faces used during training. In particular, it could have resulted from an under-representation of faces of old adults in the training sets used in machine learning^19^. This decline in performance for faces of older adults in AI could also have been boosted by the fact the possible age estimates that were produced by some of the AIs in our sample could not exceed 70 years of age. Finally, the inaccuracy in age estimation for older adults in AI compared to humans could also have been due to a larger bias in age estimation for the faces in this age group (Fig. 3c). As we noted in the Introduction, however, biases by themselves, and in particular biases due to a regression to the mean effect, cannot fully account for the pattern of errors we observed.

Still, at least in the case of human observers, it is likely that the overestimations of ages of faces of younger adults and the underestimations of faces of older adults are due to a regression to the mean effect. In particular, as in many other cases of uncertainty within a given modality^6,26^, people’s average quantitative judgments tend to be biased in the direction of the perceived, or assumed mean. The regression to the mean effect can also account for the fact that little or no bias effects were found in age estimations of faces of middle-aged adults, for which the actual age is closer to the perceived mean of the population^2,5^. Interestingly, the reduced accuracy in age estimations for female compared to male faces could also be accounted, at least in part, to effects of regression to the mean. In particular, as shown in Table 3, female faces’ ages were underestimated to a larger degree than male faces. Therefore, it is possible that decrease in accuracy for female faces resulted from larger regression to the mean effect for female compared to male faces. As for AI performance, it could be the case that at least part of the larger biases found in age estimation in AI compared to human observers could be attributed to larger effects of “regression to the mean” in AI technology. Still, to best of our knowledge, previous literature has not discussed regression to the mean in the domain of machine learning performance, although this basic statistical phenomenon would almost certainly be operating. Nevertheless, the exact nature whereby regression to the mean operates in AI needs to be explored.

The human observers in our study were young adults, which might have led to reduced accuracy in estimating the ages of older adults because of an “own-age bias”^27,28^. Previous studies, however, have shown no effects of own-age biases in the accuracy of age estimation^2,5^. For example, in a recent study, we found no advantage in age-estimation accuracy in middle-aged adult observers over young observers for faces of middle-aged adults^5^. In addition, in a comprehensive study that looked at the accuracy of age estimation in observers of different ages to faces of different ages, older adult participants did not show any advantage for faces of older adults. Instead, there was an overall decrease in their performance compared to participants from other age groups, and they were equally inaccurate in age estimations of faces from all age groups^2^. We can therefore conclude that the larger decrease in performance with faces of older adults in AI compared to humans in the current study did not benefit from the fact that our human participants were young adults. Indeed, it is likely that the differences between AI and human performance would be exaggerated even more for older human observers.

Another possible cause of inaccuracies in age estimation is the congruency between the ethnicity of the observer and that of the faces presented for evaluation^15^. Our human participants and the facial database we used were both Caucasian. In the case of AI, however, it is entirely possible that some AIs were trained on faces drawn from a variety of different ethnic groups, whereas the training sets of others might have been less diverse. This could account for some of the differences we observed within the 21 different AIs we tested as well as for overall difference between the performance of AI and the performance of our human observers. One finding, however, that cannot be easily explained by possible differences in the training sets is that AIs show a larger AES than human observers do. Recent evidence shows that the AES in humans is not affected by the ethnicity of the observers or the ethnicity of the faces presented^9^. Still, the so-called ‘own-race’ effect on other biases and inaccuracies in age estimation needs to be seriously addressed—but is well beyond the scope of the present study.

Two other facial attributes that affected the accuracy of age estimation were the expression and the gender of the face. Both humans and AIs showed an average decrease in performance for smiling compared to neutral faces, and for female compared to male faces. This decrease in performance was again larger for the AIs we tested. It is entirely possible that the larger decrease in AI performance again was a consequence of the training regimes, and in particular, from underrepresentation of smiling faces and female faces in the sets used in machine-learning training. We note that both effects cannot be accounted for by differences with respect to biases in age perception. This is indicated by the fact that differences in AI accuracy with smiling and neutral faces were observed even in situations in which there were no difference in the bias between the two expressions (i.e., for faces of old adults). In a similar vein, differences along AI accuracy between female and male faces were observed in situations in which there were no bias differences with respect to gender (i.e., for faces of young and middle-aged adults). As in other domains of automatic face recognition^19^, these results reinforce the need to adjust AI training protocols to avoid potential age estimation inequities based on age, gender, and facial expression.

Overall, across age group, gender, and facial expression in the set of photos we presented, the average accuracy in age estimation was significantly higher in human observers than in AI. The average difference in performance was a consequence of the larger inaccuracies in AI for faces of older adults, smiling faces, and female faces. It is important to note that our human observers were undergraduate students and had no explicit training (or expertise) in age estimation, whereas the AIs were a representative sample of the most prominent players in the field that have been trained to estimate age based on huge sets of face photos. Taking this into account, the average performance of the undergraduates we tested is impressive. This overall advantage for human observers is also evident in the individual scores of the human and AI participants. For all human observers and AIs taken together, the most accurate average performance was recorded for one of the human participants (see inset in Fig. 2). The larger biases and inaccuracies of AI compared to human observers suggest that current AI age-estimation technology still has a way to go before it will equal human performance.

Finally, it is important to note that in this study, we were not focused on the different architectures or training sets that particular AIs might use when tasked with estimating age. We acknowledge that the architecture and training sets will almost certainly have an effect on the biases and inaccuracies in age estimation. The question we were addressing here is whether or not the output of these AI platforms would be susceptible to the same biases and show the same inaccuracies as humans when confronted with faces that differ in age, facial expression, and gender. Our hope for this approach was two-fold. First, by documenting the performance of the range of AIs currently available, we would gain some insights into how humans perceive age. Second, that this exercise would provide some new directions for the development of more accurate and less biased AI technology. We believe that we have made some headway on both these fronts.

## Supplementary Information

Supplementary Information.

## Data Availability

All data generated or analyzed during this study are included in this published article [and its supplementary information files].
